# 115 Analyzing Temporal Trends and Outcomes Associated with High Prevalence Bacterial Infections in Burn Patients

**DOI:** 10.1093/jbcr/irac012.117

**Published:** 2022-03-23

**Authors:** Shivan N Chokshi, George Golovko, Juquan Song, Steven E Wolf, Amina El Ayadi, Alejandro A Joglar, Tsola A Efejuku, Kassandra K Corona, Maria Haseem, Sunny Gotewal, Giovanna De La Tejera, Phillip H Keys, Lyndon G Huang, Elvia L Villarreal, Shelby P Bagby

**Affiliations:** University of Texas Medical Branch at Galveston, Galveston, Texas; University of Texas Medical Branch at Galveston, Galveston, Texas; University of Texas Medical Branch, Galveston, Texas; University of Texas Medical Branch at Galveston, Galveston, Texas; University of Texas Medical Branch at Galveston, Galveston, Texas; University of Texas Medical Branch, Galveston, Texas; University of Texas Medical Branch, Galveston, Texas; University of Texas Medical Branch, Galveston, Texas; University of Texas Medical Branch, Galveston, Texas; University of Texas Medical Branch, Galveston, Texas; University of Texas Medical Branch, Galveston, Texas; University of Texas Medical Branch, Galveston, Texas; University of Texas Medical Branch, Galveston, Texas; University of Texas Medical Branch, Galveston, Texas; University of Texas Medical Branch, Galveston, Texas

## Abstract

**Introduction:**

Bacterial infections are a leading cause of complications in burn patients. However, ambiguity remains around the most common infectious etiologies and their resulting complications. Our study identifies which bacterial infections will lead to specific complications and tracks infection rates of these bacteria over time.

**Methods:**

Burn patients diagnosed with a bacterial infection within 6 months of burn were identified in the TriNetX database using ICD-10 codes; those with bacterial infections prior to injury were excluded. Occurrence of the following outcomes within 12 months of injury were compared for those with bacterial infections and those without, including acute kidney injury (AKI), congestive heart failure (CHF), hypertrophic scarring, sepsis, and death. The top 4 bacterial infections, by incidence, were then identified and analyzed for the outcomes. Lastly, infection rates were stratified by year from 2010-2020. Data was analyzed using chi-square with p < .05 considered significant, and regressions.

**Results:**

We identified 457,383 burn patients, of whom 4,688 (1.0%) were diagnosed with a bacterial infection within 6 months of injury. The bacteria that constituted the highest proportion of infected patients were *Staph aureus* (51.1%), *E. Coli* (20.2%), *Pseudomonas* (17.6%), and *Enterococcus* (9.6%). When outcomes were stratified by bacteria, *Enterococcus* infection was associated with the highest incidence of AKI (23.1%), sepsis (25.2%), and mortality (16.03%). *E. Coli* was associated with the highest incidence of CHF (17.7%) and *Pseudomonas* was associated with the highest incidence of hypertrophic scarring (13.3%). All data was found to be statistically significant (p< .05). Time trend data from 2010 to 2020 stratified by bacterial infection is displayed in Figure 1. Of note, *Pseudomonas* infection rates increased by 90% (r^2^ = 0.6717) while *E. Coli* infection rates increased by 33%. (r^2^ = 0.7223). In contrast, *Staph Aureus* infection rates have decreased since 2013. Lastly, *Enterococcus* infection rates displayed a fluctuating pattern with an increasing trend since 2017.

**Conclusions:**

Species identification of a post-burn bacterial infection is an important step in outcome management. Despite its low incidence, *Enterococcus* infection was associated with the highest incidence of AKI, sepsis, and mortality, and has displayed recent increases in infection rates. *Pseudomonas* has shown a similar increasing trend and is notable for hypertrophic scar formation.

